# Mutations in the *SPG7* gene cause chronic progressive external ophthalmoplegia through disordered mitochondrial DNA maintenance

**DOI:** 10.1093/brain/awu060

**Published:** 2014-04-10

**Authors:** Gerald Pfeffer, Gráinne S Gorman, Helen Griffin, Marzena Kurzawa-Akanbi, Emma L. Blakely, Ian Wilson, Kamil Sitarz, David Moore, Julie L. Murphy, Charlotte L. Alston, Angela Pyle, Jon Coxhead, Brendan Payne, George H. Gorrie, Cheryl Longman, Marios Hadjivassiliou, John McConville, David Dick, Ibrahim Imam, David Hilton, Fiona Norwood, Mark R. Baker, Stephan R. Jaiser, Patrick Yu-Wai-Man, Michael Farrell, Allan McCarthy, Timothy Lynch, Robert McFarland, Andrew M. Schaefer, Douglass M. Turnbull, Rita Horvath, Robert W. Taylor, Patrick F. Chinnery

**Affiliations:** 1 Wellcome Centre for Mitochondrial Research, Newcastle University, Newcastle upon Tyne, NE2 4HH, UK; 2 Institute of Genetic Medicine, Newcastle University, Newcastle upon Tyne, NE1 3BZ, UK; 3 Institute for Ageing and Health and NIHR Biomedical Research Centre for Ageing, Newcastle University, Newcastle upon Tyne, UK, NE4 5PL, UK; 4 Institute of Neurological Sciences, Southern General Hospital, Glasgow, G51 4TF, UK; 5 Academic Department of Neurosciences and University of Sheffield, Royal Hallamshire Hospital, Sheffield, S10 2JF, UK; 6 Belfast City Hospital, Belfast, UK; 7 Department of Neurology, Norfolk and Norwich University Hospital, Norwich, NR4 7UY, UK; 8 Neurology Department, Torbay Hospital, Torquay, TQ2 7AA, UK; 9 Department of Neurology, Ruskin Wing, King’s College Hospital, Denmark Hill, London, UK; 10 Institute of Neuroscience, Newcastle University, NE2 4HH, UK; 11 Newcastle Eye Centre, Royal Victoria Infirmary, Queen Victoria Road, Newcastle upon Tyne, NE1 3BZ, UK; 12 Department of Neuropathology, Beaumont Hospital, Dublin 9, Ireland; 13 Dublin Neurological Institute at the Mater Hospital and University College Dublin, Ireland

**Keywords:** chronic progressive external ophthalmoplegia, hereditary spastic paraplegia, paraplegin, mtDNA maintenance, *SPG7*

## Abstract

Despite being a canonical presenting feature of mitochondrial disease, the genetic basis of progressive external ophthalmoplegia remains unknown in a large proportion of patients. Here we show that mutations in *SPG7* are a novel cause of progressive external ophthalmoplegia associated with multiple mitochondrial DNA deletions. After excluding known causes, whole exome sequencing, targeted Sanger sequencing and multiplex ligation-dependent probe amplification analysis were used to study 68 adult patients with progressive external ophthalmoplegia either with or without multiple mitochondrial DNA deletions in skeletal muscle. Nine patients (eight probands) were found to carry compound heterozygous *SPG7* mutations, including three novel mutations: two missense mutations c.2221G>A; p.(Glu741Lys), c.2224G>A; p.(Asp742Asn), a truncating mutation c.861dupT; p.Asn288*, and seven previously reported mutations. We identified a further six patients with single heterozygous mutations in *SPG7*, including two further novel mutations: c.184-3C>T (predicted to remove a splice site before exon 2) and c.1067C>T; p.(Thr356Met). The clinical phenotype typically developed in mid-adult life with either progressive external ophthalmoplegia/ptosis and spastic ataxia, or a progressive ataxic disorder. Dysphagia and proximal myopathy were common, but urinary symptoms were rare, despite the spasticity. Functional studies included transcript analysis, proteomics, mitochondrial network analysis, single fibre mitochondrial DNA analysis and deep re-sequencing of mitochondrial DNA. *SPG7* mutations caused increased mitochondrial biogenesis in patient muscle, and mitochondrial fusion in patient fibroblasts associated with the clonal expansion of mitochondrial DNA mutations. In conclusion, the *SPG7* gene should be screened in patients in whom a disorder of mitochondrial DNA maintenance is suspected when spastic ataxia is prominent. The complex neurological phenotype is likely a result of the clonal expansion of secondary mitochondrial DNA mutations modulating the phenotype, driven by compensatory mitochondrial biogenesis.

## Abstract (precis)

Progressive external ophthalmoplegia (PEO) is a canonical feature of mitochondrial disease, but in many patients its genetic basis is unknown. Using exome sequencing, Pfeffer *et al*. identify mutations in *SPG7* as an important cause of PEO associated with spasticity and ataxia, and uncover evidence of disordered mtDNA maintenance in patients.

## Introduction

Progressive external ophthalmoplegia (PEO) is a classical presenting feature of mitochondrial disease, but the primary genetic basis has yet to be defined in a substantial proportion of patients. PEO and ptosis often occur in isolation, sometimes causing transient diplopia and significant field defects when severe, but in some patients PEO is part of a complex disorder involving both neurological and non-neurological features (Laforêt *et al.*, 1995). A skeletal muscle biopsy remains a central clinical investigation, with a mosaic pattern of cytochrome *c* oxidase (COX)-deficient fibres and ragged-red fibres (indicative of mitochondrial sub-sarcolemmal accumulation) being key diagnostic features in most, but not all cases (Taylor *et al.*, 2004).

In many patients with PEO, the underlying molecular defect is either a point mutation or a single, large-scale rearrangement of mitochondrial DNA (Moraes *et al.*, 1989). However, a large proportion of patients harbour multiple mitochondrial DNA deletions in skeletal muscle which accumulate throughout life and cause the disorder (Zeviani *et al.*, 1989; Moslemi *et al.*, 1996). Several nuclear-encoded mitochondrial genes have been shown to cause these secondary defects of mitochondrial DNA (Copeland, 2008), but the underlying nuclear gene defect is not known in ∼50% of cases. Defining the molecular aetiology of this group will have direct implications for clinical management and genetic counselling, and also lead to novel mechanistic insights.

Here we show that mutations in the spastic paraplegia 7 gene (*SPG7*), which codes for the protein paraplegin (Casari *et al.*, 1998), are an important cause of sporadic PEO with multiple mitochondrial DNA deletions presenting in mid-adult life. We demonstrate increased mitochondrial mass and hyperfused mitochondria in affected individuals, and accelerated clonal expansion of mitochondrial DNA mutations contributing to a complex neurological phenotype.

## Materials and methods

### Subjects

Whole exome sequencing was performed on eight subjects with PEO and no relevant family history who had >2% COX-deficient fibres, multiple deletions of mitochondrial DNA in skeletal muscle, and no mutation in *POLG1*, *POLG2*, *SLC25A4*, *C10orf2*, *RRM2B*, *TK2*, *OPA1* and exons 5 and 13 of *DNA2* (Ronchi *et al.*, 2013). Following our initial findings, *SPG7* was sequenced in a further 60 patients with unexplained PEO and/or multiple mitochondrial DNA deletions. Clinical details of patients with mutations are listed in Table 1. This study was approved and performed under the ethical guidelines issued by each of our institutions and complied with the Declaration of Helsinki.

**Table 1:** Clinical features and diagnostic results of patients with mutations in SPG7

Patient #	Clinical features	Age at onset (years)	Current age (years)	Affected relatives	Skeletal muscle histochemistry	Multiple mitochondrial DNA deletions	Complementary DNA change	Amino acid change	Exon	Mitochondria DNA copy number status	Reference for this mutation
**GROUP A: Compound heterozygous mutations**											
**1 M**	PEO, ptosis, proximal myopathy, mild dysphagia, ataxia, spasticity	51	66	None	30% COX-deficient / 6% RRF	LRPCR +ve	c.861dup	p.Asn288*	6	Normal	Novel
							c.1672A>T	p.Lys558*	13		van Gassen *et al.*, 2012
**2 M**	PEO, ptosis, ataxia, spasticity, dysphagia, bladder symptoms, cerebellar atrophy	Mid-40s	56	Brother of 8 M	4% COX-deficient / 2% RRF	LRPCR +ve	c.1192C>T	p.Arg398*	9	Normal	Schlipf *et al.*, 2011
							c.1529C>T	p.Ala510Val	11		McDermott *et al.*, 2001
**3 M**	Mild PEO, ptosis, eye movements restricted horizontally > vertically, hypometric saccades, lower limb proximal muscle weakness, ataxia, spasticity, mild cerebellar atrophy, mild cognitive impairment (MOCA 22/30)	47	53	None	1-2% COX-deficient fibres	LRPCR +ve (minimal changes noted)	c.1529C>T	p.Ala510Val	11	Normal	McDermott *et al.*, 2001
							c.1672A>T	p.Lys558*	13		van Gassen *et al.*, 2012
**4 F**	PEO, ptosis, proximal myopathy, ataxia, spasticity, dysphagia, dysphonia, dysarthria; optic atrophy, cerebellar atrophy	49	65	Brother	2% COX-deficient / 2% RRF	LRPCR +ve	c.2221G>A	p.(Glu741Lys)	17	Normal	Novel
							c.2224G>A	p.(Asp742Asn)	17		Novel
**5 M**	Jerky pursuits, dysarthria, ataxia, spasticity, dysdiodochokinesis, acanthocytosis, bladder symptoms, cerebellar atrophy	Late 20s	59	Brother	None; occasional intermediate fibres	LRPCR +ve	c.1053dup	p.(Gly352 Argfs*44)	8	Normal	Klebe, 2012
							c.1529C>T	p.Ala510Val	11		McDermott *et al.*, 2001
**6 M**	PEO, ptosis, ataxia, spasticity, dysarthria	u/k	66	None	3% C0X—deficient / 1% RRF	LRPCR +ve	c.1454_1462del	p.(Arg485_ Glu487del)	11	Normal	Elleuch, 2006
							c.2228T>C	p.(Ile743Thr)	17		Brugman, 2008
**7 F**	PEO, ptosis, ataxia, spasticity, proximal myopathy, moderate dysarthria, bladder symptoms	Late 20s	59	None	8% COX-deficient / 1% RRF	LRPCR -ve	c.233T>A	p.(Leu78*)	2	Normal	Arnoldi *et al.*, 2008
							c.1529C>T	p.Ala510Val	11		McDermott *et al.*, 2001
**8 M**	PEO, ptosis, ataxia, spasticity, dysphagia, bladder symptoms, cerebellar atrophy	Mid-40s	51	Brother of 2M	n.d.	n.d.	c.1192C>T	p.Arg398*	9	n.d.	Schlipf *et al.*, 2011
							c.1529C>T	p.Ala510Val	11		McDermott *et al.*, 2001
**9M**	PEO, ptosis, spastic ataxia, optic atrophy, Mild myopathy, cerebellar atrophy	Mid-60s	71	None	14% COX-deficient / 4% RRF	LRPCR +ve	c.1046insC	p.(Gly352fs*44)	8	n.d.	Klebe, 2012
							c.1529C>T	p.Ala510Val	11		McDermott *et al.*, 2001
**GROUP B: Single heterozygous mutations**											
**10F**	Ataxia, spasticity, dysarthria, dysdiadocho-kinesia, cerebellar atrophy	50	63	None	Normal	LRPCR +ve	c.184-3C>T	(splicing defect)	Splice site before Exon 2;		Novel
							c.1457G>A ^	p.(Arg486Gln) ^	11		McDermott *et al.*, 2001
**11 M**	Isolated PEO	60s	74	None	COX- deficient and RRF present	LRPCR +ve	c.1529C>T	p.Ala510Val	11		McDermott *et al.*, 2001
**12 F**	PEO, spastic ataxia	4	44	Yes	Normal	LRPCR +ve	c.1529C>T	p.Ala510Val	11		McDermott *et al.*, 2001
**13 M**	PEO, ataxia	28	90	None	3% COX- deficient fibres	LRPCR +ve	c.1529C>T	p.Ala510Val	11		McDermott *et al.*, 2001
**14 M**	Ataxia, spasticity	u/k	55	MS (maternal uncle); mother -walking difficulties	1% COX- deficient fibres	LRPCR +ve	c.1067C>T	p.(Thr356Met)	8		Novel
**15 F**	PEO	54	58		n.a.	n.a.	c.233T>A	p.(Leu78*)	2		Arnoldi *et al.*, 2008

RRF = ragged-red fibre; LRPCR = long-range polymerase chain reaction; MOCA = Montreal cognitive assessment tool; n.d. = not determined; n.a. = not available; u/k = unknown.

Note that protein alterations without RNA/protein level evidence are in brackets. RNA evidence for mutations p.288ins*, p.Arg398*, p.Ala510Val, and p.Lys588* are included in this report.

^ This variant is designated as rs111475461, has a frequency in the population of 0.02, and is of unproven pathogenicity (McDermott *et al.*, 2001; Klebe, 2012).

### Exome sequencing

Whole blood genomic DNA was fragmented to 150–200 bp by Adaptive Focused Acoustics (Covaris), end-paired, adenylated and ligated to adapters. Exonic sequences were enriched using Agilent SureSelect Target Enrichment (Agilent SureSelect Human All Exon 50 Mb kit). The captured fragments were purified and sequenced on a GAIIx platform using 75 bp paired-end reads. Bioinformatic analysis was performed using an in-house algorithm based on published tools. Sequence was aligned to the human reference genome (UCSC hg19), using NovoAlign (www.novocraft.com). The aligned sequence files were reformatted using SAMtools and duplicate sequence reads were removed using Picard. Single base variants were identified using Varscan (v2.2) and indels were identified using Dindel (v1.01). The raw lists of variants were filtered to include variants within the Sequence Capture target regions (±500 bp). On target variants were annotated using wAnnovar and common variants with a minor allele frequency > 0.02 that were present in the 1000 Genomes (February 2012 data release), the NHLBI-5400 Exome Sequencing Project and 191 unrelated in-house exomes were excluded. Rare, protein altering, homozygous and compound heterozygous variants that fitted the recessive disease model were identified.

### Sanger sequencing and multiplex ligation-dependent probe amplification analysis

Sanger sequencing of *SPG7* was performed in the entire cohort of 68 patients using custom-designed primers (http://frodo.wi.mit.edu), PCR amplification with Immolase (Bioline), and Sanger sequencing with BigDye® Terminator v3.1 (Life Technologies) according to the manufacturer’s protocol on a 3130XL Genetic Analyzer (Life Technologies), addressing regions of poor exome coverage in the eight original subjects. Exon deletions of *SPG7* were assessed by multiplex ligation-dependent probe amplification (MRC-Holland kit P089-A1) in patients with single heterozygous missense mutations. Because of the close relationship of paraplegin with AFG3L2, we also sequenced the mutational hotspots of *AFG3L2* (exons 10, 15, and 16; Cagnoli *et al.*, 2010) in patients with single heterozygous *SPG7* mutations.

### Muscle histochemistry and mitochondrial DNA analysis

Cryostat sections (10 µm) were cut from transversely orientated muscle blocks and subjected to COX, succinate dehydrogenase (SDH), and sequential COX-SDH histochemical reactions (Taylor and Turnbull, 1997). Total genomic DNA was extracted from muscle by standard procedures. Large-scale mitochondrial DNA rearrangements were screened by long-range PCR using a pair of primers (L6249: nucleotides 6249–6265; and H16215: nucleotides 16 225–16 196) to amplify a ∼10 kb product in wild-type mitochondrial DNA (GenBank Accession number NC_012920.1). The level of deleted mitochondrial DNA in individual COX-deficient and COX-positive reacting muscle fibres isolated by laser microcapture was determined by quantitative real-time PCR using the ABI PRISM® Step One real-time PCR System (Life Technologies) as previously described (He *et al.*, 2002). Furthermore, the assessment of mitochondrial DNA copy number in patient muscle was investigated by real-time PCR (Blakely *et al.*, 2008).

### Transcript expression using reverse transcription-quantitative polymerase chain reaction

Primary fibroblast cell lines were established from skin biopsies of four patients with *SPG7* mutations (Patients 1–4). Cultures were grown using minimum essential medium (Life Technologies), with 10% foetal calf serum, 2 mM l-glutamine, 50 µg/ml streptomycin, 50 U/ml penicillin, 110 mg/l Na-pyruvate and 50 mg/l uridine, trypsinized and pelleted for RNA extraction. Cells were also grown with medium supplemented with 100 µg/ml of emetine [an inhibitor of nonsense mediated messenger RNA decay; (Noensie *et al.*, 2001)] for 10 h. Cells were pelleted and RNA extracted using RNeasy® Mini Kit (Qiagen). For muscle RNA extraction, 30 mg of tissue (Patients 1–4, and three control subjects) was homogenized over ice using a Potter-type tissue homogenizer in RLT buffer (from RNeasy® Mini Kit, Qiagen) with 0.01% 2-mercaptoethanol. Homogenates were spun at 6000*g* for 5 min and supernatant used for RNA extraction as per the protocol for RNeasy® Mini Kit (Qiagen). Quality of extracted RNA was analysed using the Agilent RNA 6000 Pico Kit with an Agilent Bioanalyser 2100 (Agilent), as per the manufacturer’s instructions. Extracted RNA used in this study had a RNA integrity number ranging from 7.4–9.3.

Complementary DNA was generated using SuperScript® III reverse transcriptase kit and oligo dT primers (Life Technologies), as per manufacturer’s instructions. Transcript-specific primers for *SPG7*, *AFG3L2*, *OPA1*, *POLG*, *SDHA*, and *GAPDH* (sequences available on request) were used with SYBR® Green (Life Technologies) on an IQ5 Bio-Rad thermal cycler (Bio-Rad). Expression data were normalized to *GAPDH*. Statistical analysis was performed in Microsoft Excel using F-test: two-sample test for variances, followed by *t*-test: two sample assuming equal or unequal variances. Statistical significance was considered when *P* two-tail < 0.05. Sanger sequencing (see methods above) of complementary DNA was also performed with transcript-specific primers (sequences available upon request) to confirm the bi-allelic nature of the compound heterozygous variants.

### Western blot analysis

Muscle tissue from Patients 1, 2, 4, 5 and 7 and three control subjects (30 mg) was homogenized over ice using a Potter-type tissue homogenizer in buffer containing 250 mM sucrose, 50 mM Tris-HCl pH 7.4, 5 mM MgCl_2_ (all Sigma) and protease inhibitor cocktail tablets EDTA-free (Roche). Subsequently, Triton™ X-100 (Sigma) was added to the final concentration of 1% and samples were sonicated for 30 min on ice in a water bath sonicator. Total protein concentration was measured by means of Bradford assay. Samples (20 μg protein) were separated through 4–15% Mini-PROTEAN® TGX™ precast gels (Bio-Rad) and transferred to polyvinylidene fluoride membranes using Trans-Blot® Turbo™ transfer system (Bio-Rad). Membranes were probed with antibodies specific to SPG7 (sc-135026, Santa Cruz Biotechnology), AFG3L2 (14631-1-AP, Proteintech), OPA1 (MS995, Mitosciences), SDHA (70kDa Complex II subunit) (MS204, Mitosciences), porin (MSA03, Mitosciences), HSP60 (GTX110089, GeneTex) and GAPDH (sc-25778HRP, Santa Cruz Biotechnology), followed by species-appropriate horseradish peroxidase-conjugated secondary antibodies (Dako), using standard protocols. Protein signal was detected with Pierce ECL2 Western Blotting substrate (Thermo Scientific) and Biospectrum 500 Imaging System (UVP) as per manufacturer’s instructions. Densitometric analysis was performed using ImageJ software (National Institute of Health). GAPDH was used to normalize the results and the ratios protein of interest/GAPDH were calculated. Data represent the mean of three independent replicates. Statistical analysis was performed in Microsoft Excel using F-test: two-sample test for variances, followed by *t*-test: two sample assuming equal or unequal variances. Statistical significance was considered when *P* two-tail < 0.05.

### Mitochondrial network analysis

Cells from four *SPG7* primary fibroblast cell lines (Patients 1–4) and three control cell lines were cultured on glass bottom dishes (Willco, HBSt-3522), and mitochondria were stained using MitoTracker Red CMXRos at 0.75 nM. Live cell imaging was performed using Nikon A1R inverted confocal microscope equipped with a ×60 objective (numerical aperture = 1.40), in culture medium without phenol red and supplemented with 25 mM HEPES. Acquisitions were performed at 3% laser power, at the frame size of 512 × 512 with perfect voxel settings (*x*, *y*, *z*, 0.12 μm). Sixty-nine *z*-planes across 8 μm were captured to allow for a 3D reconstruction of mitochondrial networks from individual cells. Deconvolution and mitochondrial network analysis was performed using Huygens Essentials software (SVI). Fifty cells were imaged per individual cell line.

### Deep resequencing of mitochondrial DNA

PCR amplification of the mitochondrial DNA control region (*MT-HVS2)* in muscle DNA from six patients (Patients 1, 2, 4, 5, 6 and 7) was performed with tagged primers and ultra deep sequencing achieved using a Roche 454 GS Titanium FLX platform as previously described (Payne *et al.*, 2011). An analysis pipeline of Pyrobayes, Mosiak, and a custom R library was used to call and align the sequences to the mitochondrial DNA reference along with a control cloned mitochondrial DNA sequence. For quality control purposes, only sites covered by more than 10 000 reads in each direction were considered for analysis. Data were compared to muscle mitochondrial DNA from 22 in-house controls: seven healthy individuals undergoing orthopaedic surgery (with two being over 65 years of age), eight with recessive *POLG* mutations known to cause a high mutation burden, six with dominant *OPA1* mutations known to cause a defect of mitochondrial fusion-fission and control cloned mitochondrial DNA. Data were analysed as described previously (Payne *et al.*, 2013), with a 0.2% heteroplasmy detection threshold, based on the sequencing of a cloned mitochondrial DNA template.

## Results

### Molecular genetics

Eight patients with PEO, multiple mitochondrial DNA deletions and no known genetic defect were subjected to whole exome sequencing. After excluding common variants found in the NHLBI-5400 Exome Sequencing project, 1000 Genomes and 191 in-house disease control subjects, we identified one patient with compound heterozygous *SPG7* mutations, one of which had not been previously reported (Patient 1) and another patient with a single heterozygous mutation within the *SPG7* gene (Patient 12) (Table 1). This led us to sequence *SPG7* in the remaining larger cohort. Nine patients from eight families were found to carry compound heterozygous *SPG7* mutations, comprising three novel mutations: a stop-gain mutation, c.861dupT p.Asn288* (Patient 1); and two missense mutations c.2221G>A p.(Glu741Lys) and c.2224G>A p.(Asp742Asn) in Patient 4. Seven previously reported mutations were also identified: p.Ala510Val (six patients), p.Lys558* (two patients), p.(Leu78*) (one patient), p.Arg398* (two patients), p.(Ile743Thr) (one patient), p.(Gly352Argfs*44) (two patients) and p.(Arg485_Glu487del) (one patient). A single heterozygous *SPG7* mutation was identified in six additional patients, comprising two further novel mutations: c.184-3C>T (g.19571C>T, predicted to remove a splice site before exon 2), and c.1067C>T; p.(Thr356Met); and two previously reported pathogenic mutations: p.Ala510Val (three patients), and p.(Leu78*) (one patient) (Table 1). The most common mutation was p.Ala510Val, identified in nine patients (eight probands) from our panel of 68 probands (12%). No additional mutations or gene rearrangements were detected after multiplex ligation-dependent probe amplification analysis. No mutations in *AFG3L2* were identified.

### Clinical features of patients with SPG7 mutations

#### Compound heterozygous SPG7 mutations

**Figure 1:**
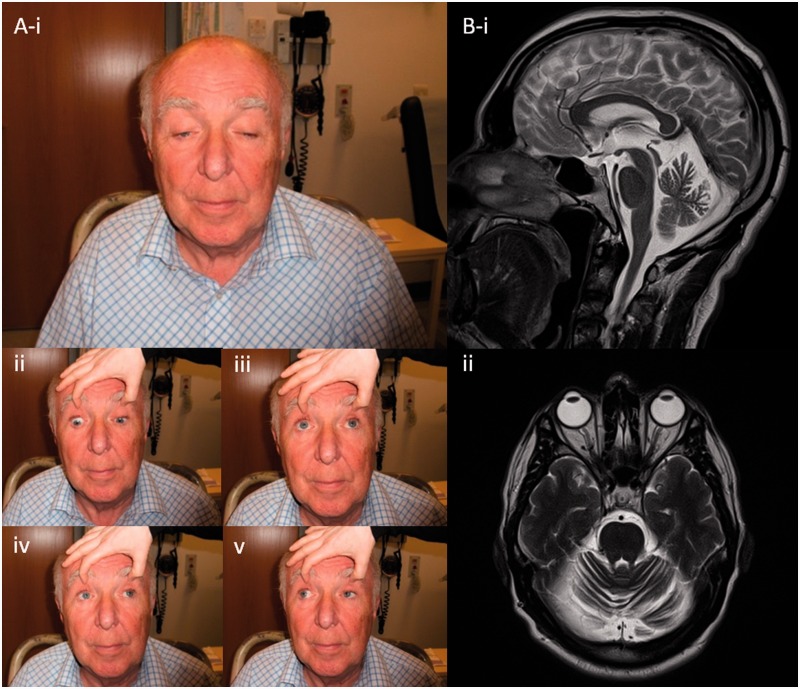
Clinical features. (**A**) Typical ophthalmological features of a patient with hereditary spastic paraplegia type 7 with PEO: marked ptosis is evident in primary gaze (i); extraocular motility in cardinal directions of gaze is mildly reduced, with restriction of upgaze most affected as the patient is asked to look down (ii); up (iii); left (iv); and right (v). (**B**) T_2_-weighted MRI images in demonstrating diffuse cerebellar volume loss, in sagittal (i); and transverse-axial (ii) planes.

**Figure 2:**
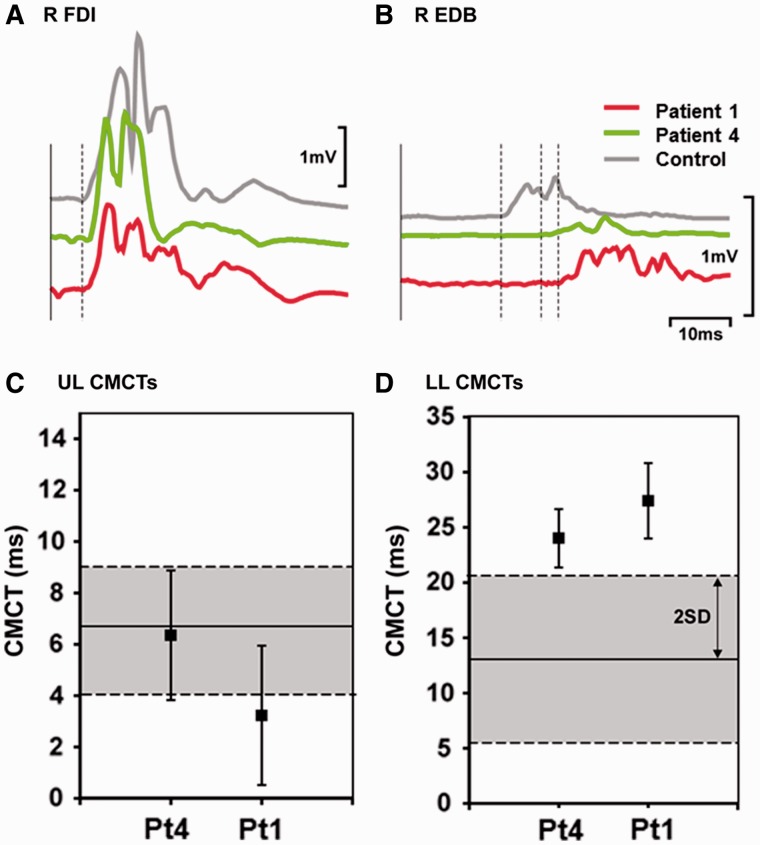
Motor evoked potentials in Patients 1 and 4. Average (*n = *10) rectified motor cortical evoked potentials (MEPs) recorded from (**A**) hand muscle, the right first dorsal interosseous (R FDI) and (**B**) foot muscle right extensor digitorum brevis (R EDB) in an age-matched male control (aged 64) shown in grey, Patient 1 (aged 65) in green and Patient 4 (aged 66) in red. Traces have been aligned after subtracting peripheral motor conduction times. Dashed lines indicate the onset of each MEP. Average central motor conduction times (mean ± 1 SD) for (**C**) right first dorsal interosseous and (**D**) right extensor digitorum brevis in the same patients. Average central motor conduction times (CMCTs) were calculated by subtracting the average peripheral motor conduction time (*n = *10) from the average motor cortical evoked potential latency (*n = *10), measured from unrectified EMG. The solid horizontal lines show the mean, dashed horizontal lines and grey shaded areas show 2 SD of the mean from published normal data (Eisen and Shtybel, 1990).

The clinical features of nine patients with compound heterozygous *SPG7* mutations are summarized in Table 1, Patients 1–9. Mean age at onset was ∼40 years (range 28–65 years) with current age 61 years (range 51–71 years). The most frequent clinical features of our patients were spastic ataxia (all nine patients) with both PEO and ptosis in eight patients (Fig. 1). Additional features included a proximal muscle weakness (five patients) and swallowing difficulties (four patients) resulting in mild to moderate disability. Other symptoms typically associated with hereditary spastic paraparesis were less frequent, including bladder dysfunction (three patients), and optic atrophy (two patients) resulting in significant visual impairment (one patient). Dysarthria was common (four patients). Other central neurological features of mitochondrial disease were not seen, such as encephalopathy, epilepsy, or stroke-like events, and cognitive impairment was observed in only a single patient (Patient 3 had a Montreal Cognitive Assessment Tool score of 22/30, losing 5 points for recall and 3 points for visuospatial). Sensorineural hearing loss was not a feature. Cardiac involvement was not evident. Cerebellar atrophy was present in all those who underwent magnetic resonance brain imaging (five patients); this was marked in four patients and mild in one patient. Motor evoked potentials performed in two patients with compound heterozygous mutations showed electrophysiological evidence of a length dependent degenerative process affecting corticospinal tracts axons projecting to the lower limb motor neurons (Fig. 2), as classically described in hereditary spastic paraplegia (Lang *et al.*, 2011).

#### Single heterozygous mutations

The clinical features of six patients with single heterozygous *SPG7* mutations are summarized in Table 1, Patients 10–15. Mean age at onset was ∼26 years (range 6–65 years) with current age 66 years (range 44–90 years). PEO (four patients) was the most common clinical feature in this group of patients and was the only finding in two patients (Patients 11 and 15). Ataxia (three patients) and other cerebellar features including nystagmus (one patient), dysdiadochokinesia (one patient) and cerebellar atrophy (one patient) were evident. Lower limb spasticity was present in three patients.

Muscle fatigue was the presenting feature in all of the patients, followed by a progressive gait ataxia with spasticity. Proximal weakness developed later in the disease course in some subjects, and PEO/ptosis was a late feature.

### Muscle mitochondrial DNA analysis

**Figure 3:**
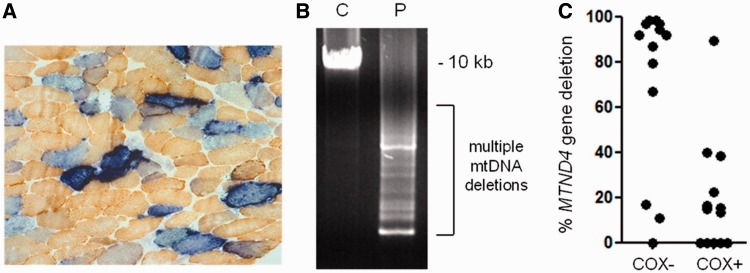
Characterization of mitochondrial DNA maintenance defect in Patient 1. (**A**) Sequential COX-SDH histochemistry demonstrates a mosaic distribution of COX-deficient muscle fibres (blue) amongst fibres exhibiting normal COX activity (brown), with significant evidence of mitochondrial proliferation as shown by enhanced SDH reactivity around the subsarcolemmal region of the muscle fibre (ragged-blue fibres). (**B**) Long range PCR amplification of muscle DNA across the major arc shows significant evidence of multiple mitochondrial DNA deletions. C = Control; P = patient. (**C**) Quantitative, single fibre real-time-PCR reveals the majority—but not all—of COX-deficient fibres contain high levels of a clonally-expanded mitochondrial DNA deletion involving the *MTND4* gene, an observation which is consistent with multiple mitochondrial DNA deletions due to a disturbance of mitochondrial DNA maintenance (He *et al.*, 2002).

Diagnostic histology and oxidative enzyme histochemistry of the patients’ skeletal muscle biopsies revealed evidence of mitochondrial respiratory chain deficiency, with sequential COX-SDH histochemistry confirming variation in the severity of the COX-mosaic defect (Table 1). These findings were particularly pronounced in Patient 1 in whom ∼30% COX-deficient fibres were noted, together with typical ‘ragged-blue’ fibres indicating subsarcolemmal mitochondrial accumulation (Fig. 3A). Long-range PCR amplification of muscle DNA clearly showed the presence of multiple mitochondrial DNA deletions (Fig. 3B), indicative of a disorder of mitochondrial DNA maintenance. Real-time PCR analysis of individual, laser-captured COX-deficient fibres showed that the majority, but not all, of these fibres harboured high levels of clonally-expanded mitochondrial DNA deletion involving the *MTND4* gene (Fig. 3C), a consistent observation in patients with genetically-proven multiple mitochondrial DNA deletion disorders (Longley *et al.*, 2006; Hudson *et al.*, 2008; Blakely *et al.*, 2012; Pitceathly *et al.*, 2012). Similar findings were also noted in Patients 2, 4, 6 and 7 (not shown). No major abnormality of mitochondrial DNA copy number was detected in muscle DNA from any of the patients with compound heterozygous *SPG7* mutations (Table 1).

### Transcript analysis

**Figure 4:**
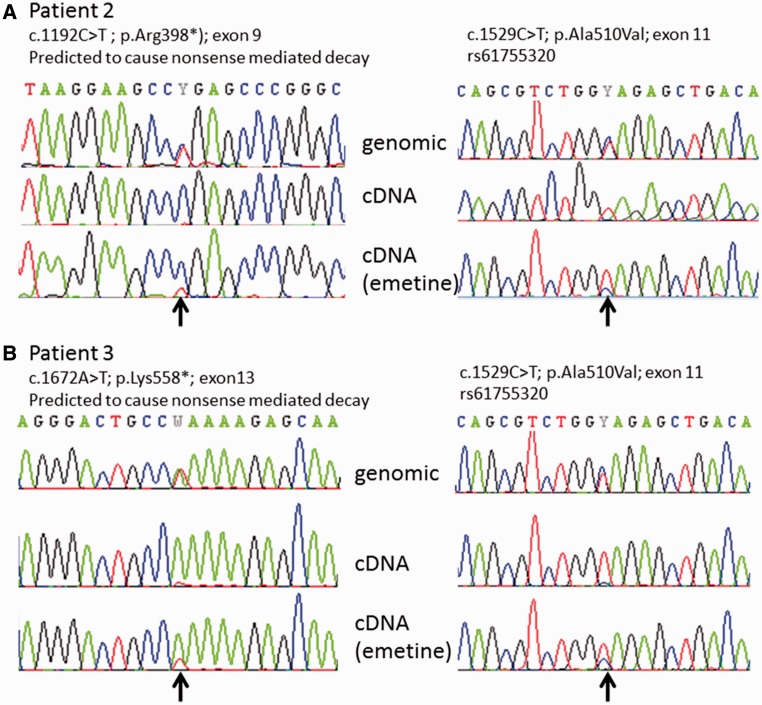
Confirmatory complementary DNA sequencing in Patients 2 and 3. Arrows indicate the positions of the mutations. (**A**) In Patient 2, the c.1192C>T (p.Arg398*) mutation is predicted to cause nonsense mediated messenger RNA decay. At left the genomic sequence demonstrates this mutation to be present in the heterozygous state although in complementary DNA from fibroblasts it appears absent because it is degraded by nonsense mediated decay. The presence of this variant is partially restored in fibroblasts grown with emetine (an inhibitor of nonsense mediated decay). The second mutation in this patient [c.1529C>T; p.(Ala510Val)] is present at homozygous levels in complementary DNA indicating that it is on the opposite allele; as before the second allele is partially restored with emetine treatment. (**B**) Patient 3 has a c.1672A>T (p.Lys558*) mutation which similarly is predicted to cause nonsense mediated decay. The mutation is almost absent in complementary DNA but partially restored in cells grown with emetine. The second c.1529C>T; p.(Ala510Val) mutation is again shown to be present on the opposite allele.

**Figure 5:**
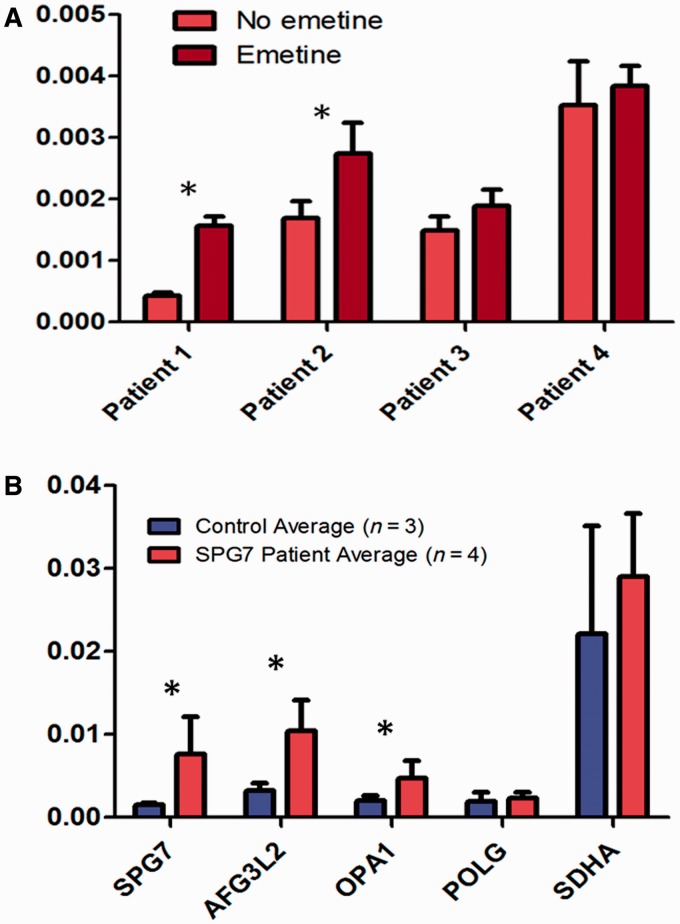
Transcript level measurement with reverse transcription quantitative PCR. (**A**) *SPG7* transcript analysis in Patients 1–4, using RNA from cultured fibroblasts. For Patients 1–3 who have one or more nonsense mutations in *SPG7*, treatment of fibroblasts with emetine (dark bars) (an inhibitor of nonsense-mediated messenger RNA decay) compared with normal conditions (light bars), resulted in significantly increased *SPG7* messenger RNA in Patients 1 and 2 (*P < 0.05). Patient 4 who has 2 missense *SPG7* mutations does not have alteration in *SPG7* expression with emetine (nor do control; data not shown). (**B**) Transcript quantitation in complementary DNA derived from muscle, in Patients 1–4 (red bands), compared with three control muscle samples (blue bands). Levels of *SPG7*, *AFG3L2*, and *OPA1* transcripts are elevated in patients compared with controls (*P < 0.02). Levels of *POLG* and *SDHA* did not differ significantly.

Sequencing of complementary DNA derived from fibroblasts in Patients 2 and 3 only revealed one mutated allele, consistent with the prediction that these two patients harboured one allele likely to cause nonsense mediated decay, and confirming that the heterozygous mutations were *in trans* (Fig. 4). In accordance with this, the transcript levels increased following emetine treatment in two of the patients with nonsense *SPG7* mutations (Fig. 5), but not in the one cell line with two missense *SPG7* mutations (Patient 4). These findings were confirmed by Sanger sequencing of complementary DNA with transcript-specific primers (Fig. 4).

Reverse-transcriptase quantitative PCR of complementary DNA derived from muscle demonstrated elevated expression of *SPG7*, *AFG3L2* and *OPA1* transcripts in patients compared with controls (Fig. 5). The transcript levels of *POLG* and *SDHA* did not differ significantly between patients and controls.

### Western blot analysis

**Figure 6:**
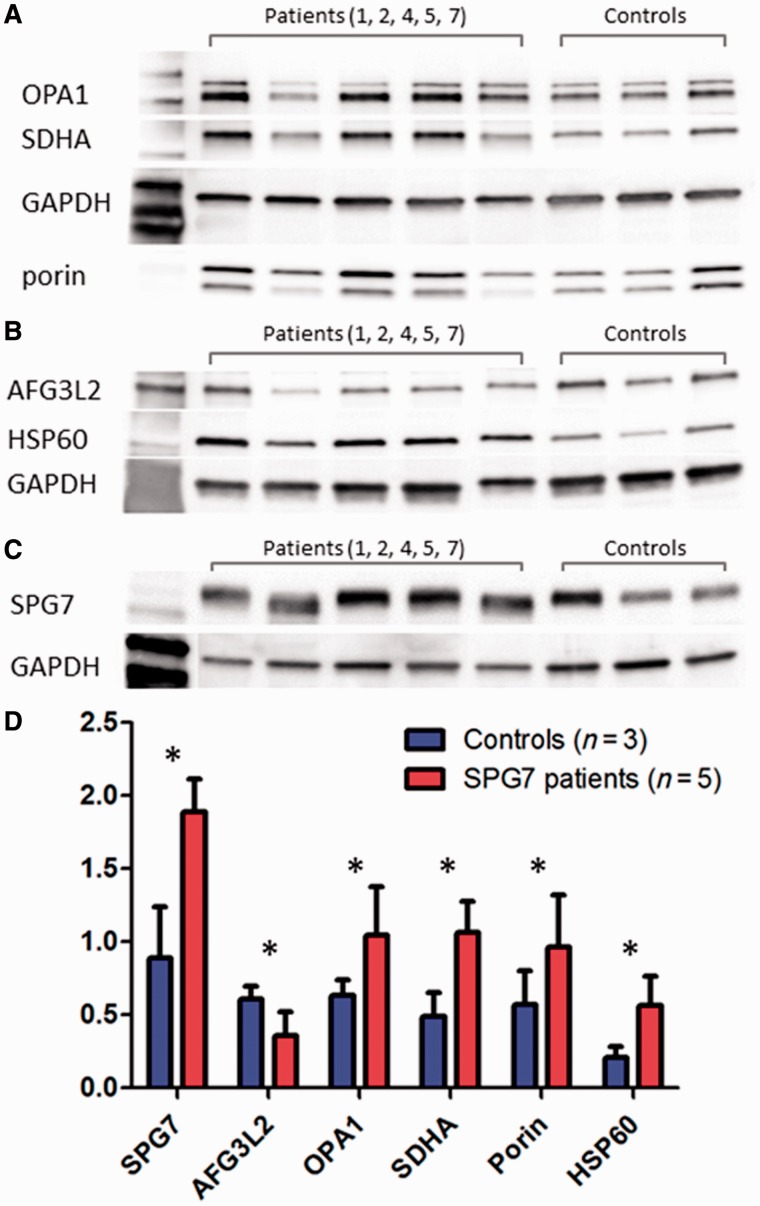
Western blot of muscle tissue. (**A–C**) Representative blots used in the quantification of protein expression of muscle tissue from five patients with compound heterozygous *SPG7* mutations and three control subjects. Testing was performed in triplicate and quantification of aggregate mean with SD (normalized to GAPDH) are represented in **D**. Markers of mitochondrial mass, including SDHA, porin, and HSP60 are significantly increased in *SPG7* patients compared with controls. SPG7 and OPA1 were also significantly elevated in *SPG7* patients compared with controls. AGF3L2 was decreased compared with controls. All the above were statistically significant to *P < 0.01.

Western blot of skeletal muscle protein showed a generalized increase in mitochondrial protein levels in the *SPG7* Patients 1, 2, 4, 5 and 7, including markers of mitochondrial mass (SDHA, porin, and HSP60) and SPG7. By contrast, AFG3L2 protein levels were reduced in patients compared to controls (Fig. 6).

### Mitochondrial network analysis

**Figure 7:**
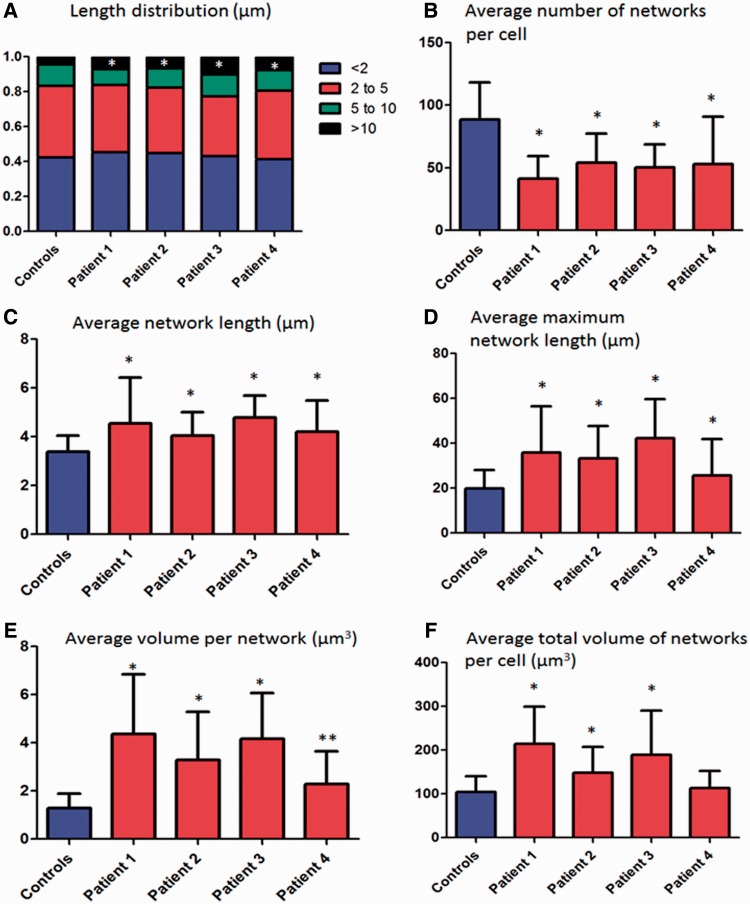
Mitochondrial network analysis. Mitochondrial network analysis was undertaken in fibroblasts (Patients 1–4) grown concurrently with identical medium and conditions. Error bars are SD. Controls are the aggregate results of four separate cell lines (50 cells each for total 200 cells). (**A**) The distribution of the network lengths is demonstrated; very long networks (>10 µm) were significantly more abundant in patients with compound heterozygous *SPG7* mutations than controls. (**B**) The average number of total networks per cell was significantly lower in *SPG7* patients. (**C**) The average length per mitochondrial network was increased in *SPG7* patients, as was the average longest network per cell (**D**). The volume of individual mitochondrial networks was higher than controls per cell line (**E**) and the total volume of the mitochondrial network per cell was elevated (in all cell lines except Patient 4 which was not significant) (**F**). We suggest that these hyperfused mitochondrial networks may be a compensatory mechanism, and that the elevated total mitochondrial volume corresponds to the elevated mitochondrial mass observed in COX-deficient fibres in these patients. *P < 0.0001 and **P = 0.004.

**Figure 8:**
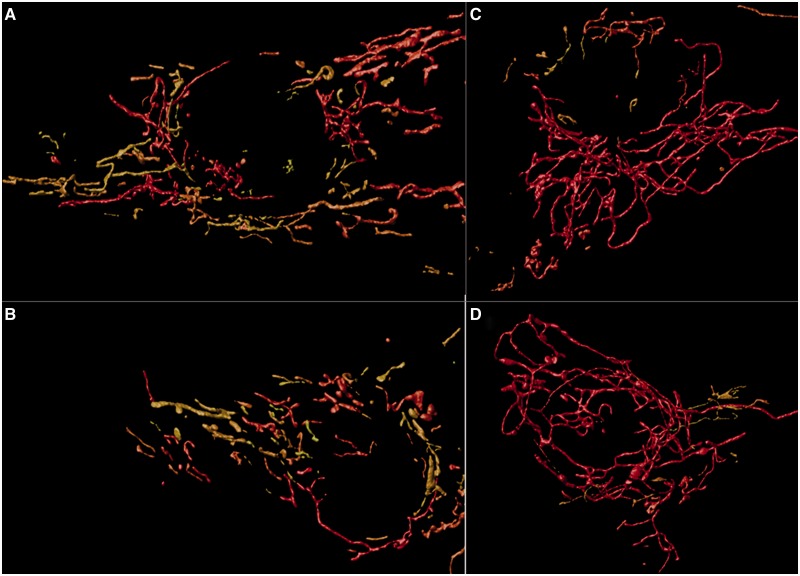
Representative images from mitochondrial network analysis. Three-dimensional reconstruction of mitochondrial networks using Huygens Essentials software. Networks are colour-coded, in which short networks are yellow and longer networks are red. (**A** and **B**) Representative images of the networks from two separate control cell lines. (**C** and **D**) Representative images from cell lines derived from Patients 1 and 4, respectively. Qualitatively, one can observe that networks appear to be longer in the patient with compound heterozygous *SPG7* mutations. Statistical analysis indicated that *SPG7* patient cell networks were on average longer, with fewer networks but increased total volume of mitochondria.

Fibroblasts from *SPG7* patients had fewer mitochondrial networks (41.42–53.90 compared with 88.66 for controls; Fig. 7), which were larger than controls. The average network length was significantly longer for *SPG7* patients (4.05–4.78 µm compared with 3.39 µm for control subjects; Fig. 7C) the average length of the longest network per cell was also significantly higher in *SPG7* patients (25.73–42.27 µm compared with 19.92 µm for control subjects; Fig. 7D), and *SPG7* patients had a greater proportion of long networks (>10 µm, 4.4% for the controls compared with 6.8 to 10.2% for the *SPG7* patients; Fig. 7A) when compared with control subjects. In addition, the average volume per mitochondrial network was greater in *SPG7* patients, as was the average total volume of mitochondrial networks per cell (Fig. 7E and F). All of these findings were highly statistically significant (*P* < 0.0001) except for average maximum network length in Patient 4 (*P* = 0.004), and no significant difference for total mitochondrial network volume per cell in Patient 4. Representative images from control and *SPG7* cell lines are presented in Fig. 8.

### Deep resequencing of mitochondrial DNA

**Figure 9:**
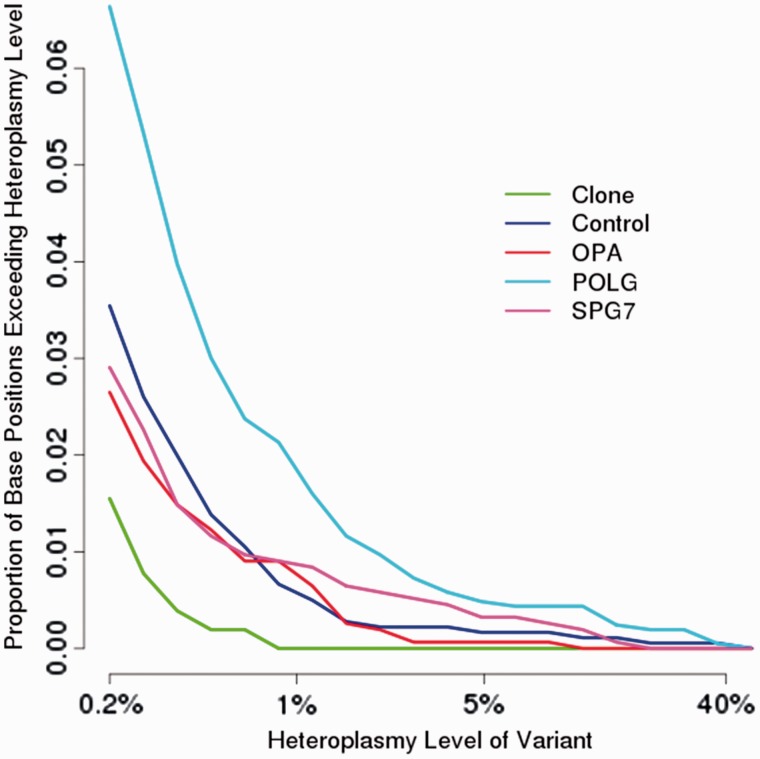
Ultra-deep resequencing of mitochondrial DNA control region. Ultra-deep resequencing by synthesis (UDS) of skeletal muscle mitochondrial DNA. UDS (Roche 454 FLX Titanium) mitochondrial DNA hypervariable segment 2 (MT-HV2) amplicon. Comparison is made between a cloned segment (expected to be homoplasmic), with controls, and patients with genetically-confirmed mitochondrial DNA maintenance disorders due to recessive *POLG*, dominant *OPA1*, or recessive *SPG7* mutations. The mutation burden in *SPG7* patients was not statistically different from control subjects or *OPA1* mutation carriers, but was significantly lower than *POLG* patients.

Analysis of FLX ultra-deep resequencing was performed on 375 positions that met our minimum criteria of >10 000-fold coverage, with 258 not associated with poly-mononucleotide repeats. Overall, the frequency of low-level mitochondrial DNA heteroplasmy (<1%) in *SPG7* patients mutations was similar to control subjects, aged controls, patients with *OPA1* mutation and lower than *POLG* patients (Fig. 9). However, the number of high-level heteroplasmies (>1%) appeared to be greater in the *SPG7* patients compared with controls or *OPA1* patients, in keeping with an increased rate of clonal expansion of mitochondrial DNA point mutations, although this difference was not statistically significant (*P* = 0.07).

## Discussion

Using an unbiased exome sequencing approach we identified pathogenic compound heterozygous *SPG7* mutations in patients with PEO and multiple mitochondrial DNA deletions in skeletal muscle, and confirmed these unexpected findings in a larger cohort of undiagnosed patients with multiple mitochondrial DNA deletions. The majority of the compound heterozygotes had at least one known pathogenic *SPG7* mutation, and both transcript and western blot analyses support a pathogenic role for the other mutations (Table 1), including novel nonsense mutations causing nonsense-mediated decay. Although we are unable to provide proof of pathogenicity for the novel mutations in Patient 4, these were associated with near-identical clinical findings to the other *SPG7* patients, and had similar abnormalities on western blot, reverse transcription quantitative PCR, and mitochondrial network imaging. Given that these mutations affect a critically important region of the protein (Bonn *et al.*, 2010), they are highly likely to be pathogenic. The presence of compound heterozygous *SPG7* mutations in these nine patients from a cohort of 68 PEO patients indicate that *SPG7* mutations are a common cause of PEO and that this gene should be sequenced in PEO patients with unexplained multiple deletions of mitochondrial DNA.

### Clinical features in patients with compound heterozygote SPG7 mutations

Given our ascertainment methods, it is not surprising that the majority of the patients with *SPG7* mutations had PEO, usually associated with marked ptosis. Although this has been previously reported in association with *SPG7* (van Gassen *et al.*, 2012), it was so uncommon that it was considered possibly a coincidental finding not related to the disorder. Our findings show that PEO and ptosis fall within the spectrum of complex *SPG7* phenotypes, and the presence of multiple mitochondrial DNA deletions provides the likely mechanism. Bladder dysfunction was seen in three patients, which has been reported in ∼50% of patients with *SPG7*-related hereditary spastic paraplegia (van Gassen *et al.*, 2012). Optic atrophy, recognized as part of a more severe *SPG7* complex phenotype (van Gassen *et al.*, 2012), was seen in two patients in our cohort resulting in significant visual impairment. Although cerebellar ataxia was a feature of all of the patients with compound heterozygous *SPG7* mutations, cortical manifestations associated with other forms of mitochondrial disease such as cognitive impairment, epilepsy, encephalopathy and/or stroke-like events were not observed. Motor evoked potentials performed in two patients showed electrophysiological abnormalities classical of hereditary spastic paraplegia, which has infrequently been reported in patients harbouring *OPA1* mutations (Yu-Wai-Man *et al.*, 2010; Baker *et al.*, 2011). This provides further evidence of corticospinal tract dysfunction and indicates that spasticity is not as rare in mitochondrial disorders as was previously thought.

### Patients with single heterozygous SPG7 mutations

Although it is possible that a second recessive *SPG7* variant is present in an area outside our analysis, perhaps in a regulatory region of the gene, dominant *SPG7* mutations have been described (Sanchez-Ferrero *et al.*, 2013), and diffusion tensor imaging demonstrated abnormalities in an asymptomatic heterozygote *SPG7* mutation carrier (Warnecke *et al.*, 2010). Furthermore, even after excluding the compound heterozygotes from our study, *SPG7* mutations remain significantly enriched in the remaining 60 patients: the common p.Ala510Val mutation is present in 3 of 118 chromosomes (2.5%) but among regionally-matched controls only 1 in 192 chromosomes (0.5%). Given that the majority of the patients with single heterozygous mutations in *SPG7* had a similar phenotype to the patients with compound heterozygous mutations, the heterozygous mutations are likely to be involved in the pathogenesis of the disorder in these patients. Although only one of six heterozygotes we studied reported a relevant family history, incomplete penetrance for presumed dominant *SPG7* mutations has been reported previously (Sanchez Ferrero *et al.*, 2013). Further familial segregation studies are warranted to definitely determine the inheritance pattern for the presumed dominant *SPG7* mutations described here.

### Novel mutations in SPG7

We demonstrate evidence of pathogenicity for three mutations (p.Asn288*, p.Arg398* and p.Lys558*) that are predicted to cause nonsense-mediated messenger RNA decay. One of these, p.Asn288*, is a novel mutation, whereas the others have been previously reported in hereditary spastic paraplegia type 7 patients (Schlipf *et al.*, 2011; van Gassen *et al.*, 2012). Our studies in fibroblasts derived from these patients demonstrate that emetine treatment increases the transcript levels. This was directly shown using reverse transcriptase quantitative PCR in Patients 1 and 2, who had increased *SPG7* transcript levels (Fig. 5A), and indirectly in Patients 2 and 3 in whom the degraded transcript was detectable with Sanger sequencing upon treatment with emetine (Fig. 4). The consistency of our findings on western blot, reverse transcriptase quantitative PCR, and mitochondrial network imaging among Patients 1–4 (and as distinguished from control subjects) is indirectly suggestive that the two novel mutations in Patient 4 [p.(Glu741Lys) and p.(Asp742Asn)] are pathogenic.

### Functional consequences of the SPG7 mutations

Several strands of evidence indicate that *SPG7* mutations induce mitochondrial biogenesis. Histochemically we observed ragged-red fibres in skeletal muscle (Fig. 3), supported by a generalized upregulation of mitochondrial proteins on western blot analysis (SDHA, porin and HSP60); and mitochondrial network analysis revealed an increased cellular mitochondrial mass in fibroblasts. Reverse transcriptase quantitative PCR of transcript levels from muscle RNA did not demonstrate elevated SDHA although other mitochondrial proteins had increased transcript levels (*SPG7*, *AFG3L2* and *OPA1*). Taken together, these findings all support upregulation of mitochondrial gene expression, protein synthesis and increased mitochondrial mass, which are typical for a mitochondrial disorder, where the organellar proliferation is thought to be a compensatory response to malfunctioning mitochondria. This increased mitochondrial biogenesis may attenuate end-organ dysfunction and explain the late onset of disease in most of our patients. The upregulation of mitochondrial proteins may also indicate an unfolded protein response caused by decreased paraplegin activity, which was demonstrated to occur in a *SPG7* RNA knockdown study in a *Caenorhabditis elegans* model (Yoneda *et al.*, 2004). It is intriguing that these findings are the mirror image of those seen in mice with mutations in *Afg3l2*, the binding partner of paraplegin, which exhibit decreased mitochondrial protein synthesis and fragmented mitochondrial networks, leading to neurodegeneration (Almajan *et al.*, 2012). This is thought to be due to the impaired metabolism of OPA1, which mediates mitochondrial fusion (Maltecca *et al.*, 2012). In contrast, in our patients with *SPG7* mutations we observed increased mitochondrial biogenesis with hyper-fused mitochondria. This again is likely to be part of a compensatory response, known as stress-induced mitochondrial hyperfusion (Tondera *et al.*, 2009). The generalized upregulation of OPA1 that we observed in skeletal muscle is likely to play a role in this response, as Opa1 isoforms are a key mediator of mitochondrial hyperfusion (Song *et al.*, 2007). It is unclear whether the paraplegin defects in these patients would have directly caused the elevated OPA1 levels, as previous work in animal models indicated that abnormal paraplegin is not sufficient to alter OPA1 metabolism (Duvezin-Caubet *et al.*, 2007; Ehses *et al.*, 2009).

How are these abnormalities linked to the secondary mutations of mitochondrial DNA present in our patients? There was no significant increase in the point mutation burden on deep sequencing of muscle mitochondrial DNA from *SPG7* patients, with similar levels to age-matched controls and *OPA1* patients, but significantly lower than in *POLG* patients (who are known to have a proofread-deficient mitochondrial DNA polymerase). The major mechanism leading to the increase in detectable mutations is therefore likely to be the segregation and clonal expansion of pre-existing deletions and point mutations, rather than an increase in the mutation rate *per se*, given that low-level mitochondrial DNA heteroplasmy seems to be a common finding in healthy individuals (Payne *et al.*, 2013). Initially this could be driven by mitochondrial biogenesis triggered by a disruption of mitochondrial quality control, in which paraplegin is intimately involved. The mitochondrial DNA replication which accompanied the biogenesis would lead to the accumulation of pre-existing mitochondrial DNA mutations. Once these mutations reach a critical level, they would lead to further biogenesis and the formation of ragged-red fibres. The combined effect would be a vicious cycle of events, leading to the accumulation of more mitochondrial DNA mutations, a COX-defect, and the subsequent PEO-phenotype.

The clonal expansion of somatic mitochondrial DNA mutations provides a common mechanism for the PEO, ptosis and myopathy seen in several mitochondrial DNA maintenance disorders, and our data suggest that the same is occurring in *SPG7.* However, it remains to be elucidated as to whether this same mechanism contributes to the motor system degeneration where a different mechanism may be operating. This also appears to be the case for *OPA1*, where the optic nerve degeneration does not appear to be mediated through clonally expanded mitochondrial DNA mutations (Yu-Wai-Man *et al.*, 2009). When taken together, these findings highlight the multiple downstream mechanisms that contribute to the clinical phenotype of ostensibly simple single-gene (monogenic) disorders. Why this should only occur in some mutation carriers remains to be determined, and it may depend upon the region of *SPG7* that is involved.

## Funding

G.P. is the recipient of a Bisby Fellowship from the Canadian Institutes of Health Research. P.F.C. is an Honorary Consultant Neurologist at Newcastle upon Tyne Foundation Hospitals NHS Trust, a Wellcome Trust Senior Fellow in Clinical Science (084980/Z/08/Z) and a UK NIHR Senior Investigator. P.F.C, R.W.T. and D.M.T. receive support from the Wellcome Trust Centre for Mitochondrial Research (096919Z/11/Z). P.F.C., R.W.T., R.H., R.M., G.S.G., and D.M.T. receive support from the Medical Research Council (UK) Centre for Translational Muscle Disease research (G0601943). R.W.T., R.M. and D.M.T. are supported by the Medical Research Council (UK) Mitochondrial Disease Patient Cohort (G0800674) and the UK NHS Highly Specialised ‘Rare Mitochondrial Disorders of Adults and Children’ Service. P.F.C. receives additional support from EU FP7 TIRCON, and the National Institute for Health Research (NIHR) Newcastle Biomedical Research Centre based at Newcastle upon Tyne Hospitals NHS Foundation Trust and Newcastle University. CLA is the recipient of a National Institute for Health Research (NIHR) doctoral fellowship (NIHR-HCS-D12-03-04). The views expressed are those of the author(s) and not necessarily those of the NHS, the NIHR or the Department of Health. MRB is funded by the NIHR, Wellcome Trust and Academy of Medical Sciences. SRJ is supported the Wellcome Trust.

## Abbreviations

COX: cytochrome *c* oxidase
PEO: progressive external ophthalmoplegia
SDH: succinate dehydrogenase
